# Bioinformatics analysis and qRT-PCR validation of iron metabolism-related genes in pediatric asthma

**DOI:** 10.1371/journal.pone.0346063

**Published:** 2026-04-15

**Authors:** Liang Liu, Yingying Sun, Yu Wang, Xiaofei Xie, Lina Wei

**Affiliations:** Department of Pediatrics, Affiliated Hospital to Changchun University of Chinese Medicine, Changchun, China; Chiba University, JAPAN

## Abstract

Pediatric asthma (PA) is a chronic airway disease with a complex etiology, and iron metabolism disorder is believed to be involved in its pathogenesis. In this study, we integrated three peripheral blood transcriptome datasets from the GEO database (GSE27011, GSE40888, GSE40732), which included 283 samples (155 in the PA group and 128 in the control group), and conducted a comprehensive analysis after normalization and batch effect correction. Differential expression analysis identified 15 iron metabolism-related differentially expressed genes (IMRDEGs), including *C19orf12, IREB2, XK, GDF15*. Functional enrichment analysis showed that these genes mainly participate in cellular energy metabolism, oxidative stress response, and regulation of iron homeostasis. A discrimination model based on machine learning algorithms isolated four key genes, with an area under the receiver operating characteristic curve of 0.69, indicating moderate diagnostic discrimination. qRT-PCR analysis of independent blood samples showed that *C19orf12* expression was upregulated in patients with PA, while *IREB2* expression was downregulated, consistent with bioinformatics analysis results. Immune infiltration analysis revealed significant differences in the proportions of memory CD4 + T cells and mast cells between high-risk and low-risk groups, suggesting that iron metabolism imbalance may contribute to asthma development via immune regulatory mechanisms. This study provides combined support from transcriptomic and experimental data for the potential role of IMRGs in PA, serving as a basis for further mechanistic research and clinical validation.

## Introduction

Pediatric asthma (PA) is a common chronic respiratory disease with a complex etiology and significant individual differences [1]. Current treatment mainly relies on inhalational corticosteroids and other anti-inflammatory drugs, which provide partial symptomatic relief. However, drug response differs significantly among different patients, with some exhibiting recurrent episodes and poor control of clinical characteristics despite treatment [2]. This difference suggests that solely relying on empirical treatment is insufficient to adequately address the diversity of the disease at the molecular level [3]. The lack of biomarker-based risk stratification and precision medicine strategies in PA has become a major bottleneck in personalized management [4]. Therefore, exploring diagnostic markers that can reflect the molecular mechanisms of the disease is of great clinical significance.

Iron metabolism homeostasis plays a key role in maintaining immune balance and redox status. Hepcidin, ferritin, and transferrin receptor work together to regulate iron absorption, storage, and circulation [5]. Disruption of the iron homeostasis causes excess free iron to promote ferroptosis and accumulate reactive oxygen species (ROS), which damage the airway epithelium and cause chronic inflammation [6,7]. Changes in iron load may affect the composition of the gut microbiota and immune response, thereby interfering with airway immune homeostasis, but this mechanism needs further validation [8]. Unlike general inflammatory responses, iron-dependent oxidative stress may specifically trigger immune imbalance in PA [9]. This suggests that dysregulated iron metabolism plays a unique role in the onset and progression of PA.

Recent transcriptomic studies have revealed the abnormal expression of several iron metabolism-related genes (IMRGs) in PA. The genes *SLC40A1* and *GDF15*, which are involved in iron transportation, storage, and regulation, are closely related to airway inflammation and oxidative stress [10,11]. However, changes in a single gene may not comprehensively account for the systemic characteristics of iron metabolism disorders. Thus, systematically assessing their roles in PA from a multi-gene perspective helps identify key regulatory nodes and establish a comprehensive diagnostic model.

In this study, we integrate multiple Gene Expression Omnibus (GEO) transcriptome datasets. We systematically analyze the expression patterns and potential biological functions of IMRGs in PA. Through differential analysis, functional enrichment, machine learning model construction, and immune infiltration assessment, we aim to identify key IMRGs with a diagnostic potential and to explore their application in early diagnosis and risk prediction. Through this research, we can provide a new theoretical basis and potential targets for molecular typing and precise intervention in PA.

## Materials and methods

### Data download

The study was conducted in accordance with the Declaration of Helsinki. As all study data were sourced from publicly available repositories, ethical approval and obtaining patient consent were not required.

We used the R package GEOquery (version 2.70.0) [12] to retrieve the GSE27011 [13], GSE40888 [14], and GSE40732 [15] PA datasets from the GEO database [16] (https://www.ncbi.nlm.nih.gov/geo/). All samples from the datasets were derived from *Homo sapiens*, with blood as the biological matrix. The microarray platforms for both GSE27011 and GSE40888 are GPL6244 (Table 1), whereas GSE40732 utilizes the GPL16025 platform. The GSE27011 dataset included 36 PA cases and 18 control cases; GSE40888 included 22 PA cases and 13 controls; and GSE40732 comprised 97 children with asthma and 97 controls. All children with asthma and their controls were included in this study.

**Table 1:** GEO Microarray Chip Information.

	GSE27011	GSE40888	GSE40732
Platform	GPL6244	GPL6244	GPL16025
Species	Homo sapiens	Homo sapiens	Homo sapiens
Tissue	Blood	Blood	Blood
Samples in PA group	36	22	97
Samples in Control group	18	13	97
Reference	PMID: 25256354	PMID: 25226851	PMID: 25769910

GEO,Gene Expression Omnibus; PA,Pediatric Asthma.

The GeneCards database [17] (https://www.genecards.org/) serves as a repository of IMRGs and offers extensive information on human genes. By searching for “iron metabolism” with the filters “Protein Coding” and “Relevance Score ≥ 2,” a total of 155 IMRGs were identified. Similarly, searching for the term “Iron Metabolism” in the PubMed database (https://pubmed.ncbi.nlm.nih.gov/) retrieved relevant literature [18]. After consolidating the results and removing duplicates, we identified 179 unique IMRGs, with detailed data available in S1 Table in S1 File.

To reduce the batch effects caused by the diversity of sources and chip platforms across the GEO datasets, we applied the combat function from the R package sva [19] (version 3.50.0) to address batch effects between the GSE27011, GSE40888, and GSE40732 datasets. This process resulted in a unified dataset comprising 155 PA and 128 control specimens. Finally, the combined GEO datasets were prepared for further analysis using R software. The Limma [20] package (version 3.58.1) was used to normalize and annotate the probe-level data. Principal component analysis [21] (PCA) was performed to evaluate the impact of batch effect removal on the expression matrices, comparing results before and after the removal. PCA serves as a dimensionality-reduction technique that extracts key features from high-dimensional datasets, enabling better visualization of the data in 2D or 3D plots and helping to reveal patterns and trends.

### Differentially expressed genes involved in iron metabolism and asthma in children

Based on sample categorization within the combined datasets, we classified the specimens into PA and control groups. The Limma package (version 3.58.1) was used to explore gene expression disparities between the two groups. We set the threshold value of |logFC| exceeding 0 and adj.P.Val (BH) < 0.05 as differentially expressed genes (DEGs). Genes with a logFC > 0 and adj.P.Val (BH) < 0.05 were classified as upregulated DEGs, while those with a logFC < 0 and adj.P.Val (BH) < 0.05 were designated as downregulated DEGs. The results derived from this differential analysis were subsequently used to generate a volcano plot using the R package ggplot2 (version 3.4.4). To identify iron-metabolism-related differentially expressed genes (IMRDEGs) pertaining to PA, all DEGs meeting the criteria of |logFC| > 0 and adj.P.Val (BH) < 0.05 from combined GEO datasets were analyzed. A comparison was made between these DEGs and IMRGs, and a Venn diagram was constructed to illustrate the outcomes, leading to the identification of IMRDEGs. In conclusion, by utilizing the findings derived from the differential analysis, a heatmap illustrating the IMRDEGs was generated using the R package pheatmap (version 1.0.12).

### Verification and correlation analysis of IMRDEGs in children with asthma

To verify the differential expression and correlation analysis of IMRDEGs in PA, we constructed a “volcano” plot comparing the expression levels of PA and control groups to examine gene expression differences between the two groups within the combined dataset. Furthermore, to assess the correlation among asthma-related DEGs in pediatric patients, we applied the Spearman correlation algorithm to analyze IMRDEG expression levels within the integrated datasets. The correlation analysis results are visualized via heatmaps using the R packages igraph (version 1.6.0) and ggraph (version 2.1.0). The absolute correlation coefficient (r-value) can be interpreted as follows: < 0.3 for weak or insignificant correlation, 0.3–0.5 for weak correlation, 0.5–0.8 for moderate correlation, and >0.8 for strong correlation.

### GO and KEGG enrichment analyses

GO analysis [22] is a widely used method for large-scale functional enrichment research, such as molecular function (MF), cell component (CC), and biological process (BP). KEGG [23,24] refers to a database widely applied for storing information on genomes, illnesses, biological pathways, and drugs. Enrichment analyses were performed for GO and KEGG pathways on IMRDEGs using the R package clusterProfiler (version 4.10.0) [25].

### Gene set enrichment analysis

GSEA [26] is a method used to assess the enrichment of genes within a predefined gene set, allowing for the identification of gene sets that are significantly associated with a particular phenotype. This analysis relies on a ranked gene table related to a particular phenotype, facilitating an understanding of the roles of genes contributing to that phenotype. In this investigation, we first ranked the genes from the combined GEO datasets according to their log2FC values. Subsequently, we used the R package clusterProfiler (version 4.10.0) to conduct GSEA on all genes within the integrated GEO datasets (combined datasets). The parameters applied during GSEA were as follows: a seed value of 2,022; 1,000 computations; a minimum gene count of 10; and a maximum of 500 per gene set. Access to the Molecular Signatures Database (MSigDB) [27] allowed us to use c2 gene sets. We employed the all.V2023.2.Hs.symbols.gmt gene set from the MSigDB database for GSEA, applying established screening criteria. Statistical significance was set at a p-value <0.05, and the false discovery rate (FDR) was controlled to ensure a q-value <0.25, employing the Benjamini-Hochberg (BH) correction approach.

### Establishment of a PA diagnostic model

We conducted logistic regression analysis on the combined GEO datasets, focusing on IMRDEGs pertinent to PA and iron metabolism. We aimed to generate diagnostic models for PA using combined GEO datasets by employing logistic regression analysis of IMRDEGs associated with iron metabolism. A p-value <0.05 serves as a critical threshold for identifying DEGs related to iron metabolism, which aids in establishing a logistic regression model. Subsequently, a forest plot illustrated the expression profiles of the IMRDEGs included in the model. Additionally, we utilized SVM [28] analysis, which is known for its high accuracy and minimal error rate, to analyze the model performance through 10-fold cross-validation. This approach further refines the selection of DEGs related to iron metabolism.

We applied the LASSO with a seed value of 500, family = ‘binomial’ and alpha = 1 as parameters, utilizing IMRDEGs in the SVM model via the R package glmnet [29]. We used 10-fold cross-validation to select lambda.min, the optimal regularization parameter that ensures a robust model performance. LASSO regression uses linear regression principles with a penalty (lambda times the coefficient) to reduce overfitting and enhance model stability. The results of the LASSO regression analysis were illustrated with diagnostic model diagrams and plots representing the variable trajectories. The resulting LASSO regression analysis provided a PA diagnostic model, with IMRDEGs serving as model genes. We computed the LASSO risk score (RiskScore) based on the risk coefficient derived from LASSO regression analysis using the following equation:


$$\text{risk}Score\;=\;\sum_{i}Coefficient\;\left({gene}_{i}\right)*mRNA\;Expression\;({gene}_{i})$$

### Validation and friends analysis of the PA diagnostic model

A predictive nomogram [30] is a visual tool that represents the functional relationships between multiple independent variables in a 2D coordinate system, facilitating the interpretation of their combined effects. We adopted the R package rms to create a nomogram from the logistic regression outcomes, showing gene relationships.

Following the LASSO regression analysis, we developed a calibration curve to assess the predictive accuracy and calibration performance of the PA diagnostic model. The R package ggDCA was also essential for creating decision curve analysis (DCA) [31] graphs, as per the RiskScore derived from integrated GEO datasets. DCA is a simple yet powerful method for assessing clinical prediction models, diagnostic assays, and molecular markers. Moreover, we adopted the R package pROC to illustrate receiver operating characteristic (ROC) curves and calculate the area under the curve (AUC) values derived from integrated GEO datasets, thereby evaluating the diagnostic efficacy of the LASSO risk score (RiskScore) for PA incidence.

The PA cohort was divided into high- and low-risk groups based on the median RiskScore from the diagnostic model. To investigate the differential expression of model genes between the two groups, a box plot was constructed to illustrate the expression levels of such genes. Following this, we utilized the R package pROC to create an ROC curve for model genes and compute the AUC, allowing us to evaluate the diagnostic performance of their expression levels in relation to PA. The AUC values for the ROC curve ranged from 0.5 to 1, with values near 1 implying high classification accuracy; those ranging from 0.5 to 0.7 implying low accuracy; those ranging from 0.7 to 0.9 implying moderate accuracy; and those >0.9 implying excellent robustness.

Semantic assessment of GO [22] annotations offers a quantitative approach to determine the degree of similarity between genes and genomes, which is essential for various bioinformatics investigations. We employed the R package GOSemSim (version 2.28.0) [32] to compute and analyze the functional relationships among the model genes based on their functional similarity (Friends).

### Gene set enrichment analysis for high- and low-risk groups

We stratified the combined GEO datasets into high- and low-risk cohorts according to the median LASSO RiskScore and performed differential expression analysis utilizing the R package Limma, with DEGs identified based on the criteria |logFC| > 0 and adjusted p-value (adj.p) <0.05. We categorized genes exhibiting logFC > 0 and adj.p < 0.05 as upregulated genes and those with logFC < 0 and adj.p < 0.05 as downregulated genes. The outcomes of the differential analysis were used to create a volcano plot using the R package ggplot2 (version 3.4.4). Additionally, the top 20 DEGs, arranged in descending order according to |logFC|, were used to create a heatmap using the R package pheatmap (version 1.0.12).

We initially organized the genes from the PA specimens derived from the combined GEO datasets according to the logFC values, separating the high-risk from the low-risk groups. Subsequently, the R package clusterProfiler (version 4.10.0) [25] was used to perform GSEA on a complete set of genes within the combined GEO datasets. The GSEA parameters were established as follows: a seed value of 2,020, a minimum threshold of 10 genes for each gene set, and a maximum limit of 500 genes per gene set. Access to the c2 gene sets was obtained through MSigDB, specifically all.V2023.2.Hs.Symbols, for enrichment analysis. Gene set selection in GSEA required adj.p < 0.05 and FDR value (q-value) <0.25, with p-value adjustments using the BH method.

### Construction of a regulatory network

Transcription factors (TFs) play a pivotal role in gene expression regulation by interacting with target genes (mRNA) during post-transcriptional processes. TFs sourced from the ChIPBase [33] database were integrated to conduct a thorough analysis of their regulatory influence on the model genes. To enhance the reliability of the findings, we applied specific filtering criteria, retaining only those TF-mRNA interaction relationships in which the sum of ‘Number of samples found (upstream)’ and ‘Number of samples found (downstream)’ exceeded 15. Thereafter, we visualized the mRNA-TF regulatory network using Cytoscape software.

MicroRNAs (miRNAs) play crucial roles in the regulatory mechanisms governing the development and evolution of various organisms. These small noncoding RNA molecules can modulate various target genes, and it is noteworthy that multiple miRNAs may affect a single target gene. To explore the relationship between model genes and miRNAs, we retrieved pertinent miRNAs related to model genes from the StarBase database [34] and applied filtering criteria to enhance reliability: only those miRNA-mRNA interaction relationships in which the sum of the Number of samples found (upstream) and Number of samples found (downstream) exceeded 4 were included. We then visualized the mRNA-miRNA regulatory network using the Cytoscape software to improve clarity.

### Immune infiltration analysis of disease controls (CIBERSORT)

CIBERSORT [35] utilizes linear support vector regression to deconvolute the transcriptome expression matrix. This approach enables the estimation of the abundance and composition of immune cell types in mixed cellular samples. This algorithm, when applied alongside the LM22 feature gene matrix, effectively filters out data points with an immune cell enrichment score >0, resulting in the generation of an immune cell infiltration matrix derived from the combined datasets. Comparative plots illustrate the disparities in the abundance of immune cell infiltration between the PA and control groups within these datasets. Furthermore, we employed the R package pheatmap to construct correlation heatmaps, which provide a visual representation of correlation analysis outcomes among various immune cells, as well as between model genes and immune cells. The correlation coefficients were classified as follows: absolute value <0.3 indicated a weak/negligible correlation; 0.3–0.5 indicated a weak correlation; 0.5–0.8 indicated a moderate correlation; and values between 0.8 and 1.0 indicated a strong correlation.

### Immune infiltration analysis of high- and low-risk groups (CIBERSORT)

We employed the CIBERSORT algorithm in conjunction with an immune cell signature gene matrix. Data with immune cell enrichment scores exceeding zero were subsequently filtered, yielding a detailed matrix of immune cell infiltration specific to PA specimens derived from the GEO datasets. A proportion bar chart was constructed for visual representation. Subsequently, the association between immune cells was evaluated using Spearman’s correlation coefficient. We constructed a correlation heatmap using the R package pheatmap (version 1.0.12) to visually represent the findings of the correlation analysis of immune cell interactions. Furthermore, we surveyed the correlation between the model genes and immune cells using the Spearman method and constructed a correlation bubble plot using the R package ggplot2 (version 3.4.4) to illustrate the correlation analysis outcomes between the model genes and immune cells.

### Quantitative real-time PCR (qRT-PCR) analysis

To experimentally validate bioinformatically identified core candidate genes, we performed SYBR Green-based qRT-PCR on peripheral blood samples obtained from patients with PA (n = 12) and healthy controls (n = 8). Total RNA was extracted using TRIzol reagent (Aidela, RN0102), and its quality was verified spectrophotometrically (A260/A280 = 1.8–2.1). cDNA was synthesized from 1–2 μg RNA using the SuperScript III Reverse Transcriptase kit (EXONGEN, A502). qPCR reactions were conducted on a Longgene Q2000B system with the following protocol: 95°C for 5 min; 40 cycles of 95°C for 10 s, 58°C for 20 s, and 72°C for 20 s; followed by a melt curve analysis to confirm amplification specificity. Each sample was run in triplicate, and β-actin was used as the internal control. Relative gene expression was calculated using the 2^ − ΔΔCt method, and statistical significance (p < 0.05) was determined using an unpaired Student’s t-test. Primer sequences are listed in S1 Text in S1 File.

### Statistical analysis

In this investigation, data handling and analytical procedures were performed using R software (version 4.2.2). An independent Student’s t-test was employed to evaluate the statistical significance of continuous variables following a normal distribution between two distinct groups unless stated otherwise. For abnormally distributed variables, we employed the Mann–Whitney U test and the Wilcoxon rank sum test to assess disparities. We applied the Kruskal–Wallis test for comparisons involving three or more groups. Furthermore, we performed Spearman’s correlation analysis to ascertain the correlation coefficients among the various molecular entities. Unless otherwise specified, all p-values were two-sided, with a significance threshold of <0.05.

## Results

### Technology roadmap

**Fig 1:**
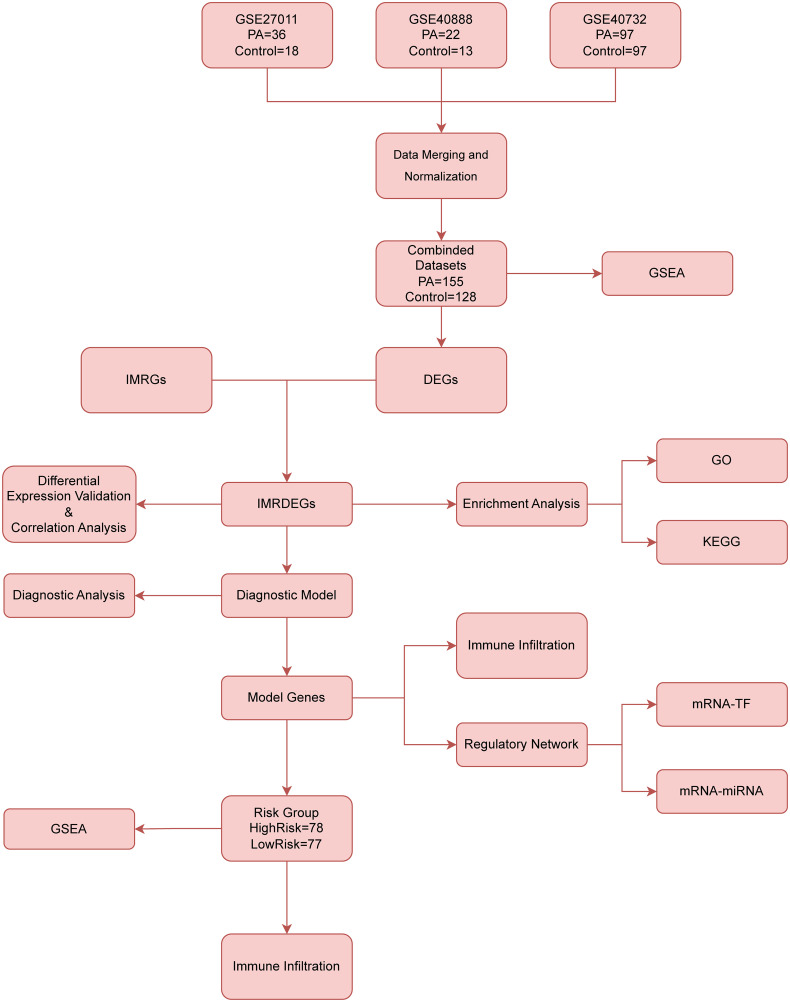
Flowchart for comprehensive analysis of IMRDEGs. PA: Pediatric asthma, TF: Transcription factor, DEGs: Differentially expressed genes, KEGG: Kyoto Encyclopedia of Genes and Genomes, IMRGs: Iron-metabolism-related genes, GO: Gene ontology, GSEA: Gene Set Enrichment Analysis, IMRDEGs: Iron-metabolism-related differentially expressed genes.

The methodology for the comprehensive analysis of IMRDEGs is summarized in the flowchart (Fig 1), which outlines the study design and data analysis workflow. The sample information of the datasets involved in this flowchart is shown in Table 1.

### Merging of PA datasets

**Fig 2:**
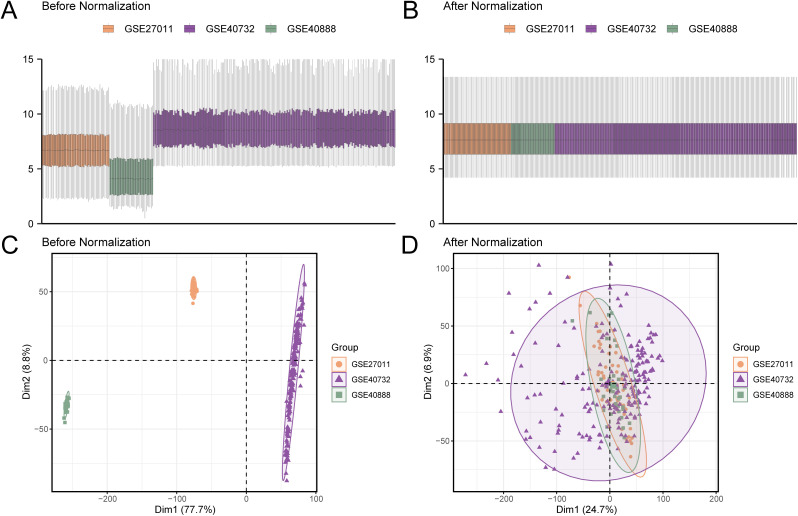
Batch effect removal in combined GEO datasets. (A). Box plots showing the distribution of the combined GEO datasets before batch removal. (B). Boxplots depicting the distribution of the same combined datasets after batch effect removal, illustrating the improved homogeneity across the datasets. (C). PCA plot showing the distribution of the datasets prior to batch effect correction. (D). PCA plot of the integrated combined GEO datasets following batch effect removal, demonstrating the improved clustering and reduced technical variation. In the PCA plots, the PA datasets are represented as follows: GSE27011 (orange), GSE40888 (green), and GSE40732 (purple). PCA: Principal component analysis, PA: Pediatric asthma, GEO: Gene Expression Omnibus.

An integrated GEO dataset was obtained by performing batch effect correction from the PA datasets (GSE27011, GSE40888, and GSE40732). We then compared the expression value disparities between the datasets before and after batch effect removal using box plots (Figs 2A–B). Additionally, we compared the low-dimensional feature distributions of the datasets before and after batch effect removal using PCA plots (Figs 2C–D). These visualizations confirmed the successful removal of batch effects, resulting in improved dataset homogeneity and clustering.

### Analysis of DEGs related to iron metabolism in PA

**Fig 3:**
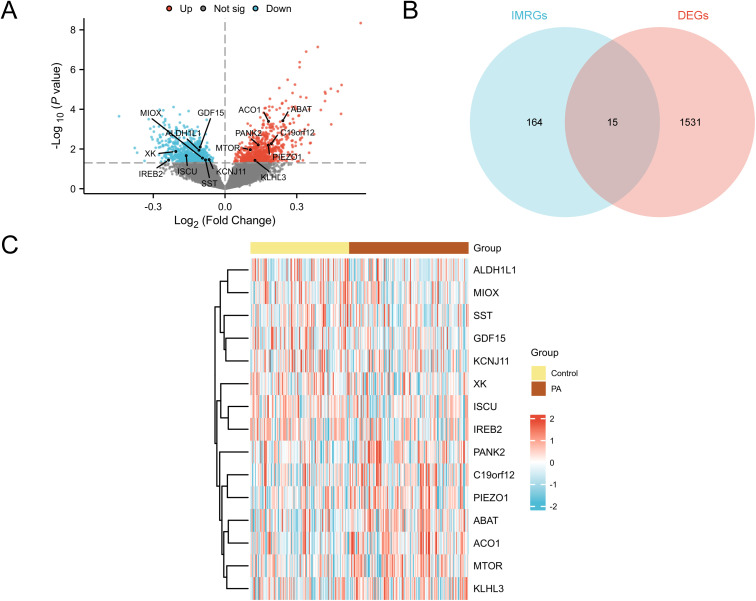
Differential gene expression analysis. (A). Volcano plot of differentially expressed genes (DEGs) analysis between control and PA groups in the combined GEO datasets. (B). Venn diagram of DEGs and iron metabolism-related genes (IMRGs) in integrated GEO datasets (combined datasets). (C). Heatmap of iron-metabolism-related differentially expressed genes (IMRDEGs) sorted by logFC in integrated GEO datasets (combined datasets). Yellow represents the control group (control), and brown represents the PA group. Red in the heatmap denotes high expression, and blue denotes low expression. Not sig.: Not significant, GEO: Gene Expression Omnibus, logFC: log fold change.

Comparative analysis between the two groups identified 775 upregulated and 771 downregulated genes. A volcano plot (Fig 3A) depicts these DEGs, with significantly upregulated and downregulated genes highlighted. To obtain IMRDEGs, the intersection of the identified DEGs and IMRGs was obtained, resulting in a Venn diagram (Fig 3B), which revealed 15 IMRDEGs, namely *ABAT*, *ACO1*, *C19orf12*, *PANK2*, *PIEZO1*, *GDF15*, *MTOR*, *ALDH1L1*, *XK*, *ISCU*, *MIOX*, *KCNJ11*, *SST*, *IREB2*, and *KLHL3*. A heatmap (Fig 3C) visualizes their expression patterns across samples.

### Verification and correlation analysis of differentially expressed iron metabolism-related genes in PA

**Fig 4:**
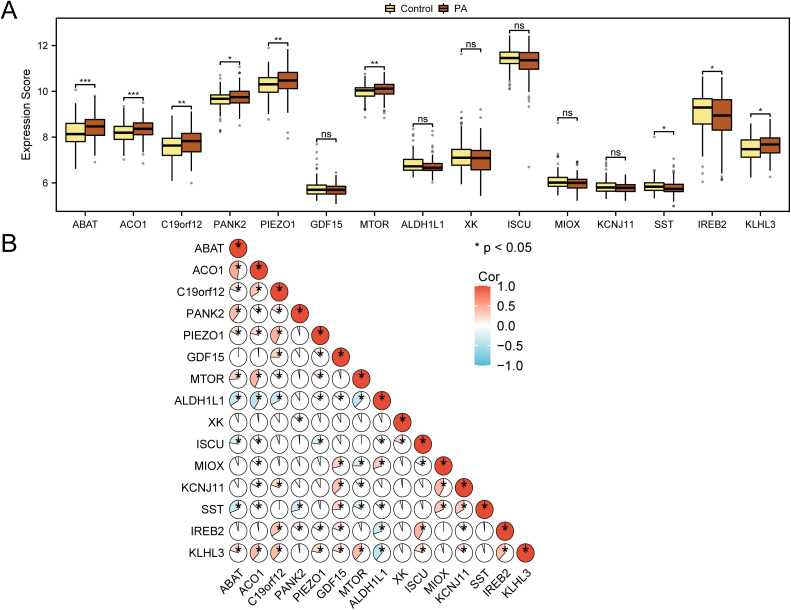
Correlation analysis of IMRDEGs. (A). Group comparison map of expression disparities in iron-metabolism-related differentially expressed genes (IMRDEGs) in the combined GEO datasets. (B). Correlation heatmap of 15 iron metabolism-related differentially expressed genes (IMRDEGs) in integrated GEO datasets (combined datasets). ns indicates no statistical significance (p ≥ 0.05); *p < 0.05; **p < 0.01; ***p < 0.001. An absolute correlation coefficient (r-value) of <0.3 indicated weak or no correlation, whereas an r-value of 0.3–0.5 indicated weak correlation. Brown denotes the PA group, and yellow denotes the control group. Red indicates a positive correlation, and blue represents a negative correlation. PA: Pediatric asthma, IMRDEGs: Iron metabolism-related differentially expressed genes, Cor: Correlation, GEO: Gene Expression Omnibus.

As illustrated in the group comparison figure (Fig 4A), differential expression analysis revealed 15 IMRDEG expression profiles, contrasting the PA group with the control group. To evaluate IMRDEG expression in the integrated GEO datasets, group comparisons revealed significant expression differences in genes such as *ABAT* and *ACO1* (p < 0.001), among others (Fig 4A). Furthermore, the expression levels of the IMRDEGs *C19orf12*, *PIEZO1,* and *MTOR* also demonstrated statistically significant variations (p < 0.01) between the PA and control groups. The expression levels of IMRDEGs *PANK2*, *SST*, *IREB2,* and *KLHL3* were statistically significant (p < 0.05) in the same comparative analysis. Correlation heatmap (Fig 4B) revealed predominantly positive correlations among the 15 IMRDEGs, suggesting their potential co-regulation.

### GO and KEGG enrichment analyses of DEGs related to iron metabolism

**Fig 5:**
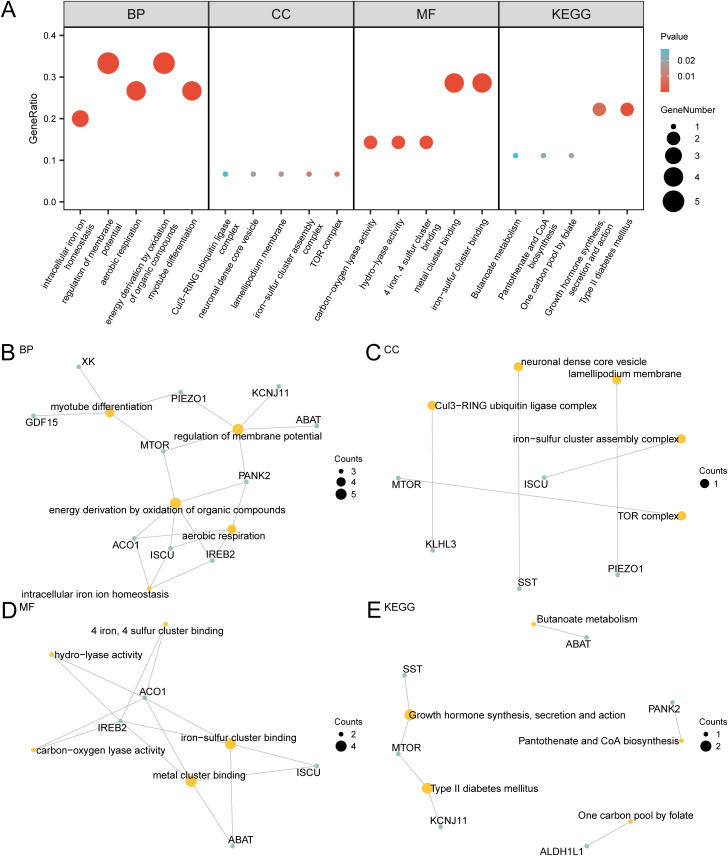
GO and KEGG enrichment analysis for IMRDEGs. (A). Bubble diagram of gene ontology (GO) and pathway (KEGG) enrichment analysis of differentially expressed genes related to iron metabolism (IMRDEGs): biological pathway (KEGG), molecular function (MF), cellular component (CC), and biological process (BP). The abscissa shows GO and KEGG terms. (B–E). GO and pathway (KEGG) enrichment analysis outcomes of the iron metabolism-related differentially expressed gene (IMRDEGs) network diagram showing BP (B), CC (C), MF (D), and KEGG (E). Yellow nodes denote items, green nodes denote molecules, and the lines denote the relationships between items and molecules. The bubble size in the bubble plot indicates the number of genes, with the bubble color reflecting the p-value. A deeper red color denotes a smaller p-value, whereas a deeper blue color indicates a larger p-value. A p-value <0.05 and FDR value (q-value) <0.25 served as screening criteria for GO and pathway (KEGG) enrichment analysis. IMRDEGs: Iron metabolism-related differentially expressed genes, CC: Cellular component, KEGG: Kyoto Encyclopedia of Genes and Genomes, GO: Gene Ontology, BP: Biological process, MF: Molecular function, FDR: false discovery rate.

Table 2 shows the main enrichment analysis results of 15 IMRDEGs related to respiratory system or immune functions in GO and KEGG, and the remaining results can be found in S2 Table in S1 File. Bubble plots (Fig 5A) illustrate the enrichment results, highlighting key pathways and processes.

**Table 2:** Results of GO and KEGG Enrichment Analysis for IMRDEGs.

ONTOLOGY	ID	Description	GeneRatio	BgRatio	pvalue	p.adjust	qvalue
BP	GO:0009060	aerobic respiration	4/15	197/18870	1.44E-05	2.76E-03	1.47E-03
BP	GO:0042391	regulation of membrane potential	5/15	440/18870	1.67E-05	2.76E-03	1.47E-03
BP	GO:0006879	intracellular iron ion homeostasis	3/15	73/18870	2.44E-05	3.24E-03	1.73E-03

GO, Gene Ontology; BP, Biological Process; CC, Cellular Component; MF, Molecular Function; KEGG, Kyoto Encyclopedia of Genes and Genomes; IMRDEGs, Iron-Metabolism-Related Differentially Expressed Genes.

Based on the above results, we drew network diagrams for the BP, CC, MF, and biological pathways (Figs 5B–E). In the diagram, the lines denote the corresponding molecules and entries, with larger nodes representing more associated molecules. Notably, the BP of energy generation via the oxidation of organic compounds and regulation of membrane potential exhibited the most substantial molecular enrichment.

### Gene set enrichment analysis

**Fig 6:**
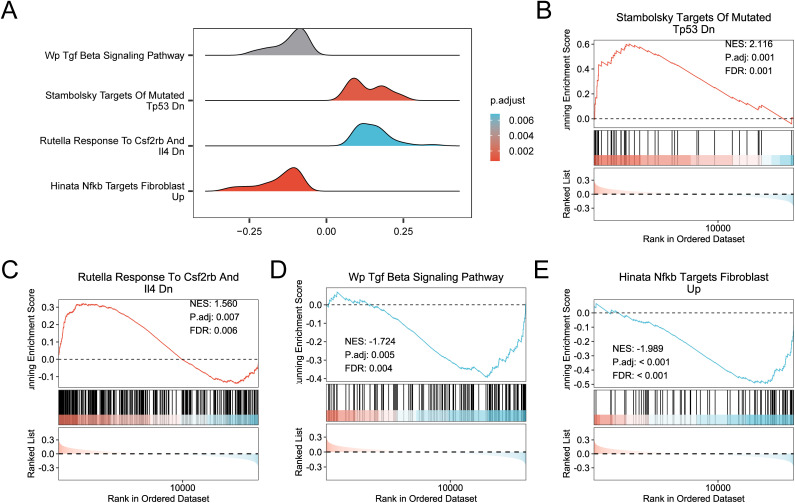
GSEA for combined datasets. (A). Gene set enrichment analysis (GSEA) mountain map presentation of biological functions in the integrated Gene Expression Omnibus (GEO) datasets (combined datasets). (B–E). GSEA results showed significant enrichment for all genes in the following pathways: Stambolsky Targets of Mutated TP53 DN (B), Rutella Response to CSF2RB and IL4 DN (C), TGF β signaling pathway (D), and Hinata NFKB targets fibroblast up (E). In the mountain map, the color represents the adjusted p-value (adj.p-value): darker red indicates a smaller adj.p-value, and darker blue indicates a larger adj.p-value. In the bubble plot, the bubble size represents the gene set size, and the bubble color indicates the adj.p-value, with darker red corresponding to smaller values and darker blue corresponding to larger values. Red in the heatmap denotes high expression, whereas blue denotes low expression. GSEA screening criteria were false discovery rate (FDR) value (q-value) <0.25 and adj.p-value <0.05, with Benjamini-Hochberg (BH) as the p-value correction method.

GSEA assessed the global expression influence in PA. Results are shown in S3 Table in S1 File. Table 3 presents key information on abnormal immune system activation, intercellular interactions, and inflammatory mediator production in PA. Fig 6A shows the link between the affected cellular components and molecular functions. Figs 6B–E present the enrichment of Strambolsky targets of mutated TP53 DN, Rutella response to CSF2RB and IL4 DN, TGF β signaling pathway, and Hinata NFKB targets fibroblast up, respectively. These pathways are closely linked to immunomodulatory signaling cascades, indicating that IMRGs may participate in the pathogenesis of PA by regulating these signaling pathways.

**Table 3:** Results of GSEA for Combined Datasets.

ID	setSize	enrichmentScore	NES	pvalue	p.adjust	qvalue
CURSONS_NATURAL_KILLER_CELLS	18	0.87	2.52	3.04E-09	7.77E-07	6.45E-07
BROWNE_INTERFERON_RESPONSIVE_GENES	60	0.61	2.35	4.18E-08	7.01E-06	5.82E-06
REACTOME_IMMUNOREGULATORY_INTERACTIONS_BETWEEN_A_LYMPHOID_AND_A_NON_LYMPHOID_CELL	110	0.55	2.31	1.45E-10	5.67E-08	4.71E-08
EINAV_INTERFERON_SIGNATURE_IN_CANCER	25	0.68	2.13	5.37E-05	2.38E-03	1.97E-03
STAMBOLSKY_TARGETS_OF_MUTATED_TP53_DN	40	0.60	2.12	2.50E-05	1.33E-03	1.11E-03
WP_EICOSANOID_SYNTHESIS	18	0.72	2.10	1.77E-04	5.68E-03	4.72E-03
BOSCO_INTERFERON_INDUCED_ANTIVIRAL_MODULE	72	0.53	2.09	2.89E-06	2.46E-04	2.04E-04
HECKER_IFNB1_TARGETS	87	0.51	2.09	9.22E-07	9.32E-05	7.74E-05
DAUER_STAT3_TARGETS_DN	41	0.58	2.05	7.07E-05	2.96E-03	2.46E-03
RUTELLA_RESPONSE_TO_CSF2RB_AND_IL4_DN	286	0.32	1.56	2.27E-04	6.82E-03	5.66E-03
WP_TGF_BETA_SIGNALING_PATHWAY	127	−0.39	−1.72	1.45E-04	5.01E-03	4.16E-03
HINATA_NFKB_TARGETS_FIBROBLAST_UP	77	−0.49	−1.99	1.09E-05	7.10E-04	5.89E-04

GSEA, Gene Set Enrichment Analysis.

### Establishment of the PA diagnostic model

**Fig 7:**
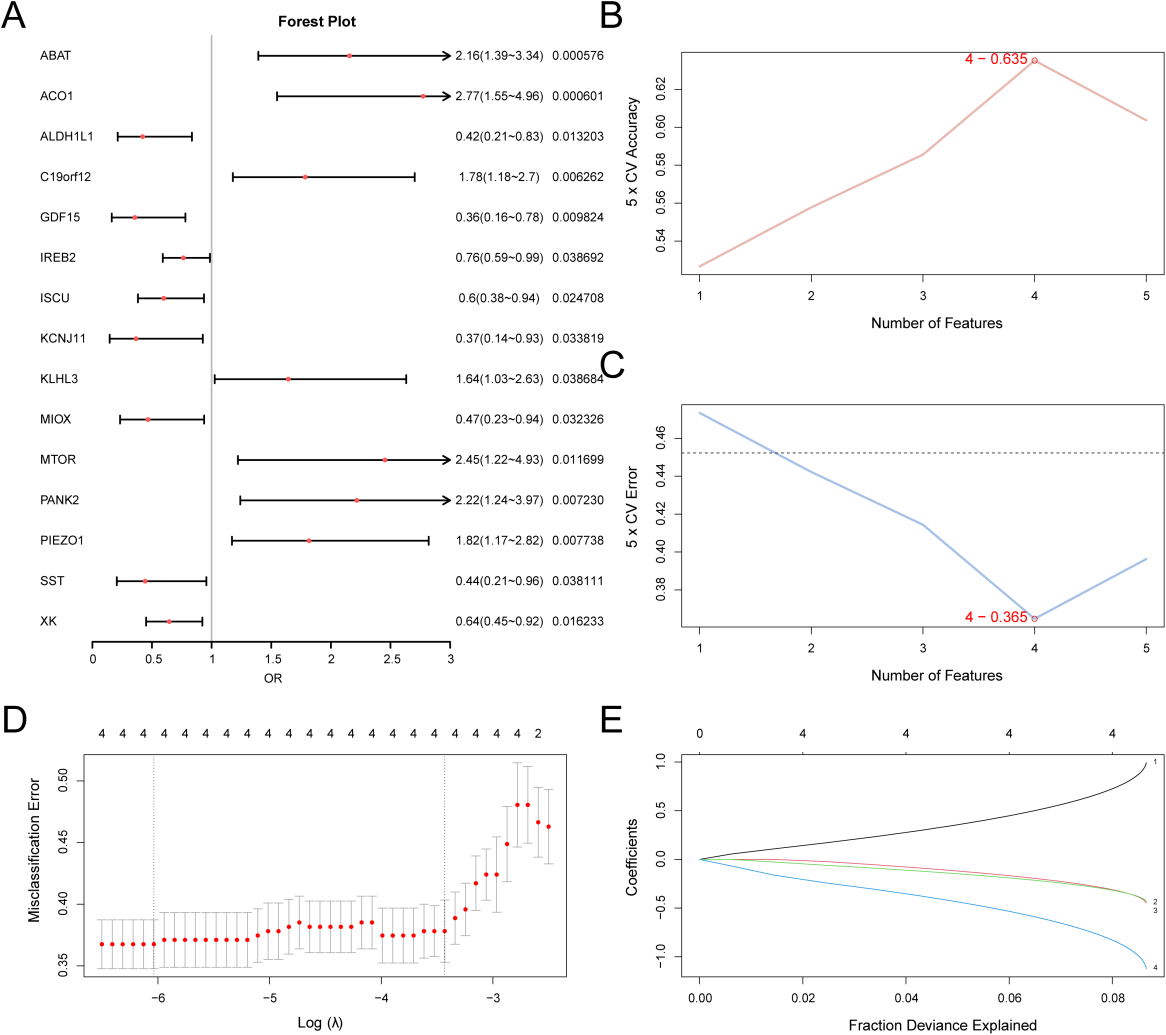
PA diagnostic model. (A). The diagnostic value of the 15 IMRDEGs included in the logistic regression model for PA is visualized through a forest plot. The horizontal axis for each gene represents the odds ratio and its 95% confidence interval; the red dots indicate the p-value of the gene, reflecting its statistical significance in the model. (B). The trend of accuracy changes in the SVM model. As the number of genes increases, the accuracy gradually rises, with the best accuracy being observed for four genes (0.6835). (C). Changes in the trend of error rates. As the number of features (genes) increases, the error rate gradually decreases, indicating that the model performs optimally when four genes are selected, with the error rate reaching its lowest point (0.365). (D). Visualization of the LASSO regression model. The error changes of the model under different regularization parameters (λ) are depicted, with the error decreasing as λ increases; the optimal λ value is marked as the one that minimizes the cross-validation error. €. The coefficient changes of each gene in the LASSO regression model. Different colors represent different genes; as the λ increases, the coefficients of each gene gradually decrease, illustrating the feature selection process. This figure demonstrates the importance of the 15 IMRDEGs in the diagnosis of PA and indicates the diagnostic potential of four key genes (C19orf12, IREB2, XK, and *GDF15*). PA: Pediatric asthma, LASSO: Least Absolute Shrinkage and Selection Operator, IMRDEGs: Iron Metabolism-Related Differentially Expressed Genes, SVM: Support vector machine.

To construct the PA diagnostic model, we initially applied logistic regression to the 15 identified IMRDEGs. A forest plot (Fig 7A) was used to graphically represent the resulting logistic regression model. This analysis confirmed that all 15 IMRDEGs within the logistic regression model were statistically significant (p < 0.05). Subsequently, 15 IMRDEGs and the SVM algorithm were employed to develop the SVM model. Figs 7B and C present the changes in the accuracy and error rate distribution of the SVM model with different numbers of genes, helping us to determine the optimal gene combination. Figs 7D and E depict the LASSO regression path and cross-validation error, supporting the stability and representativeness of the selected model genes (*C19orf12*, *IREB2*, *XK*, and *GDF15*). These illustrations reflect the rationality and scientific nature of the model construction process.

### Validation and friends analysis of the PA diagnostic model

**Fig 8:**
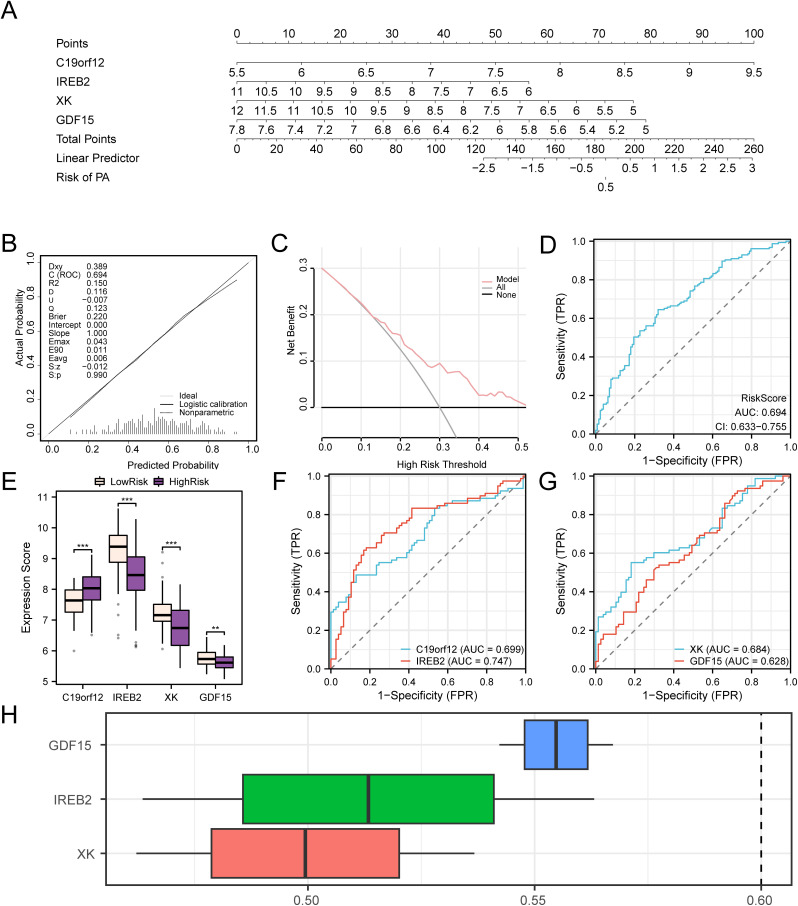
PA diagnostic and validation analysis. (A). Nomograms of the combined Gene Expression Omnibus (GEO) datasets of model genes in PA diagnostic models. (B–C). Calibration curve plot (B) and decision curve analysis (DCA) plot (C) of pediatric asthma (PA) diagnostic model based on the RiskScore in integrated GEO datasets (combined datasets). (D). ROC curve of RiskScore in the integrated GEO datasets (combined datasets). (E). Comparative charts of model genes in the high-risk and low-risk PA groups. (F–G). ROC curves of model genes C19orf12 and *IREB2* (F), *XK* and *GDF15* (G) in the PA group. (H). Boxplot of functional similarity (Friends) analysis outcomes of model genes. The ordinate of the DCA plot represents the net benefit, whereas the abscissa denotes the probability threshold or threshold probability. **p < 0.01; ***p < 0.001. The AUC demonstrated some precision, ranging from 0.7 to 0.9. Pink represents the low-risk group, whereas purple represents the high-risk group. PA: Pediatric asthma, ROC: Receiver operating characteristic, AUC: Area under the curve, DCA: Decision curve analysis.

To assess the efficacy of the PA diagnostic model, integrative relationships among these genes within the combined GEO datasets were illustrated via nomograms constructed from the model genes (Fig 8A). The findings revealed that the diagnostic value of the model gene *C19orf12* was markedly superior to that of other variables in the PA diagnostic model. Conversely, the expression of *IREB2* demonstrated significantly lower utility than the other variables within the same diagnostic framework.

Next, we evaluated the precision and resolution of the PA diagnostic model using a calibration curve. The model’s predictive performance was evaluated by comparing the actual probabilities with the predicted probabilities under various conditions (Fig 8B). The calibration curve for the PA diagnostic model shows that the red calibration line slightly deviates from the ideal diagonal but remains closely aligned. The clinical predictability of the PA diagnostic model was further assessed using DCA (Fig 8C). These findings show that the model’s line consistently remains above both the “all positive” and “all negative” lines within a certain range, indicating that the model provides a significant net benefit and demonstrates good performance. Additionally, the ROC curve demonstrates (Fig 8D) that the risk score expression level showed a certain degree of discrimination ability across the different groups (AUC = 0.69). The formula for calculating the risk score is as follows:


riskScore =Cxcl1*(2.22)+Atf3*(2.06)+Fos*(−0.47)+Ccl2*(0.39)

The PA group was subsequently categorized into high- and low-risk groups according to the median RiskScore obtained from the PA diagnostic model. Fig 8E shows the differential expression of the four model genes between these groups. These findings implied (Fig 8E) that the expression levels of the model genes *C19orf12*, *IREB2,* and *XK* differed significantly between the high- and low-risk groups (p < 0.001). Furthermore, the model gene *GDF15* expression level exhibited a high statistical significance between the two groups (p < 0.05). ROC curves were generated using the R package pROC based on the model gene expression levels in the PA group (Figs 8F–G). These results indicate that the expression levels of the model genes *C19orf12*, *IREB2*, *XK,* and *GDF15* in the PA group displayed a certain degree of precision across different groups (0.9 > AUC > 0.7).

This was confirmed by the ROC curve (Figs 8F–G), which demonstrated a notable degree of precision for these genes across the groups (0.9 > AUC > 0.7).

Functional similarity (Friends) analysis (Fig 8H) ranked *GDF15* as the most relevant gene to PA pathogenesis, being closest to the cutoff value (0.60).

### Gene set enrichment analysis for high- and low-risk groups

**Fig 9:**
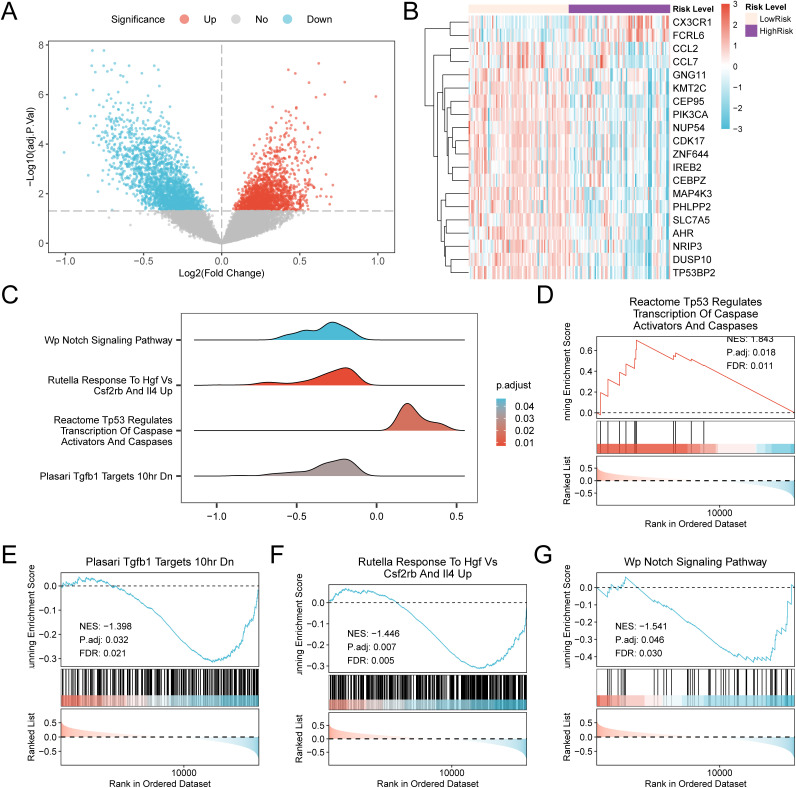
Differential gene expression analysis and GSEA according to risk group. (A–B). Volcano map (A) and heatmap of expression values (B) of differentially expressed genes analysis in high-risk and low-risk groups in the combined Gene Expression Omnibus (GEO) datasets. (C). Mountain plot presentation of four biological functions from gene set enrichment analysis (GSEA) of pediatric asthma (PA) specimens from integrated GEO datasets (combined datasets). (D–G). GSEA results indicated that PA specimens were significantly enriched in Reactome TP53 Regulates Transcription of Caspase Activators and Caspases (D), Plasari TGF-β Targets 10hr DN (E), Rutella Response to Hgf Vs Csf2rb and IL4 Up (F), and Wp Notch Signaling Pathway (G). Pink denotes the low-risk group, whereas purple denotes the high-risk group. In the mountain map, the color represents the adjusted p-value (adj.p-value): darker red indicates a smaller adj.p-value, and darker blue indicates a larger adj.p-value. In the bubble plot, the bubble size represents the gene set size, and the bubble color indicates the adj.p-value, with darker red shades corresponding to smaller values and darker blue shades corresponding to larger values. Red in the heatmap denotes high expression, whereas blue denotes low expression. Gene set enrichment analysis (GSEA) screening criteria were adj.p-value <0.05 and FDR value (q-value) <0.25, along with Benjamini-Hochberg (BH) as the p-value correction method.

Using the R package Limma for differential analysis of the combined GEO dataset, we obtained DEGs for the high- and low-risk groups. We identified a total of 4,166 DEGs, with 1,902 upregulated genes and 2,264 downregulated genes. A volcano plot (Fig 9A) was created as per the transcriptomic comparison results of such a dataset. Additionally, a heatmap was constructed to display the results of the top 20 DEGs sorted by |logFC| in descending order (Fig 9B).

To evaluate the influence of the expression levels of all genes in the combined GEO datasets on the incidence of PA, we employed GSEA using the logFC values of all genes, comparing the high- and low-risk groups. This analysis explored the associations between gene expression profiles in the integrated GEO datasets (combined datasets) and the biological processes, cellular components, and molecular functions involved, as represented in the mountain plot (Fig 9C; see S4 Table in S1 File for the detailed outcomes). Table 4 shows the pathways that are significantly associated with respiratory and immune functions, which are related to viral infections, immune regulation, epithelial functions, and key signal transduction. Figs 9D–G presents the four pathways that were significantly enriched in the high-risk group, namely, TP53-mediated transcriptional regulation of apoptotic factors, downregulation of TGF-β target genes, enhanced immune response related to hepatocyte growth factor, and activation of the Notch signaling pathway. The enrichment of these pathways further supports the possibility of stronger immune activation and tissue remodeling tendencies in the high-risk group.

**Table 4:** Results of GSEA for Risk Group.

ID	setSize	enrichmentScore	NES	pvalue	p.adjust	qvalue
BLANCO_MELO_RESPIRATORY_SYNCYTIAL_VIRUS_INFECTION_A594_CELLS_DN	96	0.54	2.32	8.12E-10	3.15E-08	2.03E-08
BOSCO_EPITHELIAL_DIFFERENTIATION_MODULE	57	0.52	2.11	7.33E-06	1.10E-04	7.10E-05
SANA_RESPONSE_TO_IFNG_UP	62	0.52	2.11	3.58E-06	5.96E-05	3.84E-05
REACTOME_TP53_REGULATES_TRANSCRIPTION_OF_CASPASE_ACTIVATORS_AND_CASPASES	11	0.70	1.84	3.76E-03	1.77E-02	1.14E-02
PLASARI_TGFB1_TARGETS_10HR_DN	235	−0.32	−1.40	8.18E-03	3.23E-02	2.08E-02
RUTELLA_RESPONSE_TO_HGF_VS_CSF2RB_AND_IL4_UP	370	−0.31	−1.45	1.25E-03	7.37E-03	4.75E-03
WP_NOTCH_SIGNALING_PATHWAY	52	−0.43	−1.54	1.32E-02	4.62E-02	2.97E-02

GSEA, Gene Set Enrichment Analysis.

### Construction of a regulatory network

**Fig 10:**
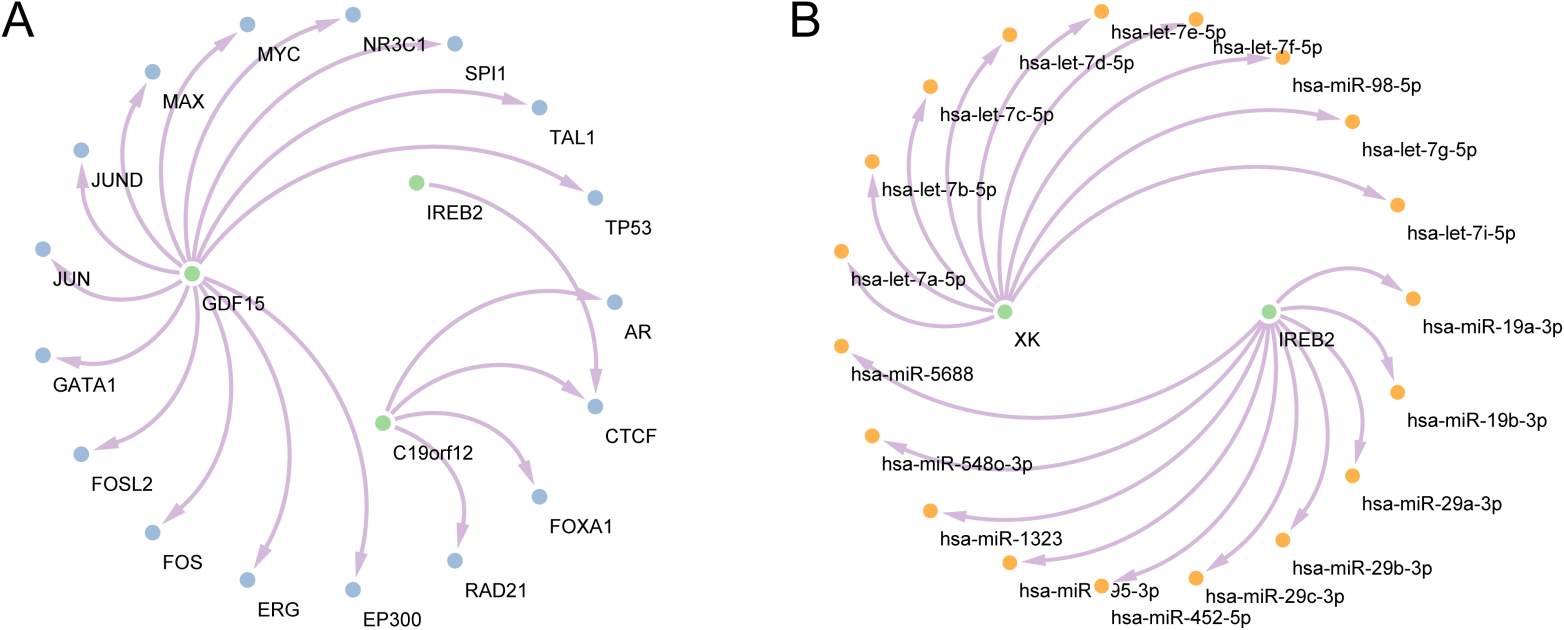
Regulatory network of model genes. (A). mRNA-TF regulatory network of model genes. (B). mRNA-miRNA regulatory network of model genes. Green denotes mRNA, blue denotes TF, and yellow denotes miRNA. TF: Transcription factor.

To explore the possible upstream transcriptional regulatory mechanisms of the model genes, a regulatory network of these genes was constructed. Initially, TFs related to model genes were retrieved from the ChIPBase database, facilitating the construction and visualization of the mRNA-TF regulatory network via the Cytoscape software (Fig 10A). This network comprised three model genes and 17 TFs, with elaborate data provided in S5 Table in S1 File. Subsequently, miRNAs connected to the model genes were sourced from the StarBase database, resulting in the development and visualization of the mRNA-miRNA Regulatory Network through the Cytoscape software (Fig 10B). This network includes two model genes and 19 miRNAs, with specific details available in S6 Table in S1 File.

### Immune infiltration analysis of the disease control group (CIBERSORT)

**Fig 11:**
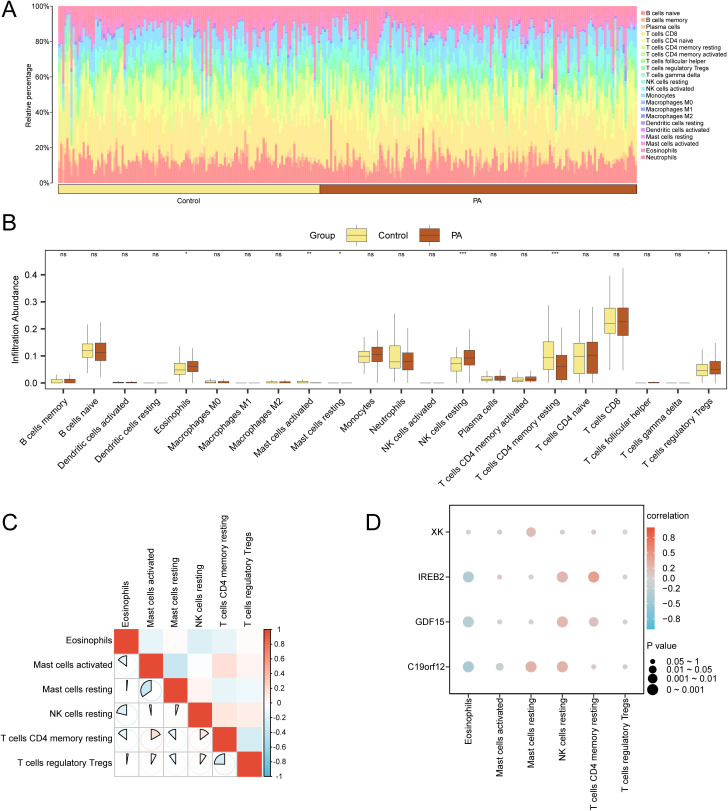
Combined dataset immune infiltration analysis by the CIBERSORT algorithm. (A–B). The proportion of immune cells in integrated Gene Expression Omnibus (GEO) datasets (combined datasets) is shown as a bar graph (A) and group comparison graph (B). (C). Correlation heatmap illustrating relationships among immune cells in integrated GEO datasets (combined datasets). (D). Correlation bubble plots showing the association between immune cell infiltration abundance and model genes in combined GEO datasets. ns indicates no statistical significance (p ≥ 0.05); *p < 0.05; **p < 0.01; ***p < 0.001. An absolute correlation coefficient (r-value) of <0.3 indicated weak or no correlation, 0.3–0.5 indicated weak correlation, 0.5–0.8 indicated moderate correlation, and >0.8 indicated strong correlation. Yellow represents the control group, and brown represents the PA group. Red denotes a positive correlation, whereas blue denotes a negative correlation. Color depth reflects correlation strength. PA: Pediatric asthma.

The immune infiltration abundances of the 22 immune cell types within the combined GEO datasets were evaluated using the CIBERSORT algorithm. Initially, a bar chart representing the proportion of immune cells in the integrated GEO datasets was generated based on the results of immune infiltration analysis (Fig 11A). Group comparison charts showed differences in immune cell abundance among the various groups. The analysis (Fig 11B) revealed statistically significant disparities (p < 0.05) in all six immune cell types, including resting memory CD4 + T cells, regulatory T cells (Tregs), resting natural killer cells, resting mast cells, activated mast cells, and eosinophils.

Furthermore, correlation outcomes regarding the abundance of the six immune cell types in the immune infiltration analysis of PA specimens were illustrated using a correlation heatmap (Fig 11C). These findings revealed strong correlations between most immune cells, with the most significant negative correlation observed between resting and activated mast cells (r = –0.346, p < 0.05). Finally, the correlation between the model genes and immune cell infiltration was visualized using a correlation bubble plot (Fig 11D), which revealed substantial correlations between most immune cells. Notably, the analysis revealed a strong positive correlation (r = 0.416, p < 0.05) between *IREB2* and resting memory CD4 + T cells.

### Immune infiltration analysis of high- and low-risk groups (CIBERSORT)

**Fig 12:**
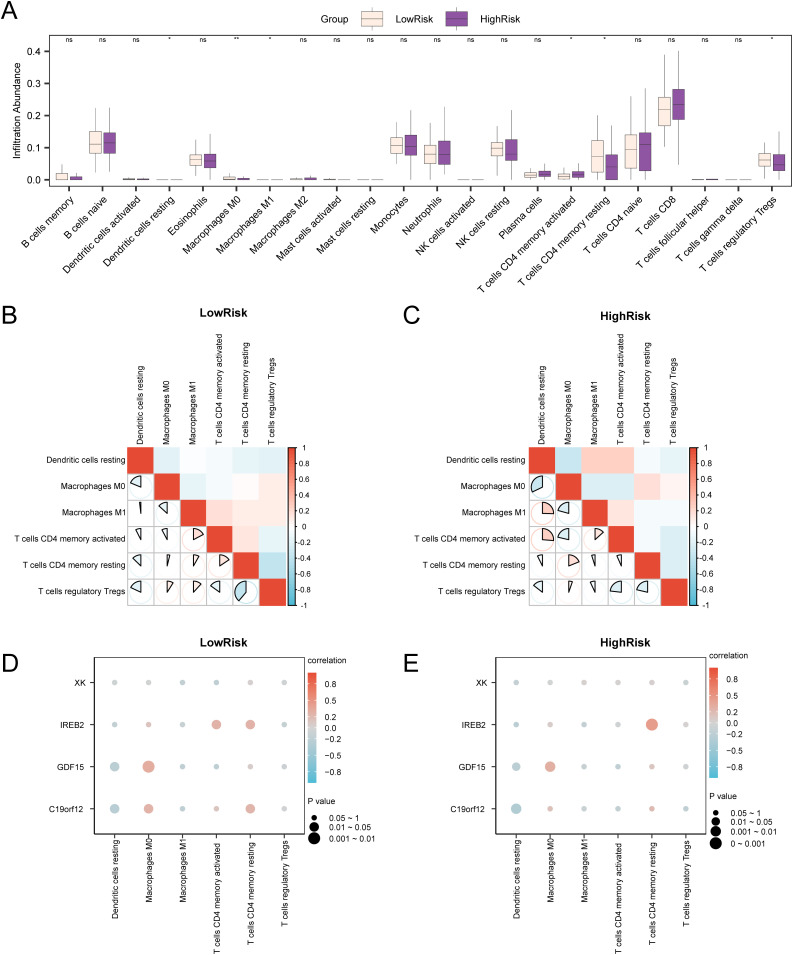
Risk group immune infiltration analysis by the CIBERSORT algorithm. (A). Comparison of immune cells in low- and high-risk PA groups. (B–C). Heatmap of the correlation between immune cells in the low-risk (B) and high-risk (C) groups of pediatric asthma (PA) specimens. (D–E). Bubble plot of the correlation between immune cell infiltration abundance and model genes in the low-risk (D) and high-risk (E) groups of PA specimens. An absolute correlation coefficient (r-value) of <0.3 indicated weak or no correlation, 0.3–0.5 indicated weak correlation, 0.5–0.8 indicated moderate correlation, and >0.8 indicated strong correlation. Pink indicates the low-risk group, whereas purple indicates the high-risk group. Red denotes a positive correlation, whereas blue denotes a negative correlation. Color depth reflects correlation strength.

The abundances of 22 immune cell types were quantified using the CIBERSORT algorithm on PA specimens derived from the combined GEO datasets. Initially, we illustrated the immune infiltration analysis findings using a relative proportion stacked plot, which displayed variations in the relative composition of immune cell infiltration among different cohorts. A stacked column chart (Fig 12A) indicated that all six immune cell types assessed were statistically significant (p < 0.05), specifically memory resting CD4 + T cells, memory activated CD4 + T cells, Tregs, M0 macrophages, M1 macrophages, and resting dendritic cells.

Subsequently, the abundance correlations of these six immune cell types were represented using a correlation heatmap (Figs 12B–C). These findings suggested that most immune cells in the low-risk PA group exhibited strong correlations, with the most pronounced negative correlation observed between memory resting CD4 + T cells and Tregs (r = –0.388, p < 0.05) (Fig 12B). Conversely, in the high-risk group, most immune cells also demonstrated significant correlations, with M0 macrophages and resting dendritic cells exhibiting the strongest negative correlation (r-value = –0.322, p < 0.05) (Fig 12C). Lastly, the relationship between model genes and immune cell infiltration abundance was depicted in correlation bubble plots (Figs 12D–E). The results from these plots indicate that, in the low-risk group, most immune cells exhibited strong correlations, particularly highlighting a notable positive correlation between the *GDF15* gene and M0 macrophages (r = 0.307, p < 0.05) (Fig 12D). We found the most substantial positive correlation between the *IREB2* gene and memory resting CD4 + T cells (r = 0.434, p < 0.05) (Fig 12E) in the high-risk group.

### qRT-PCR validation of candidate genes

**Fig 13:**
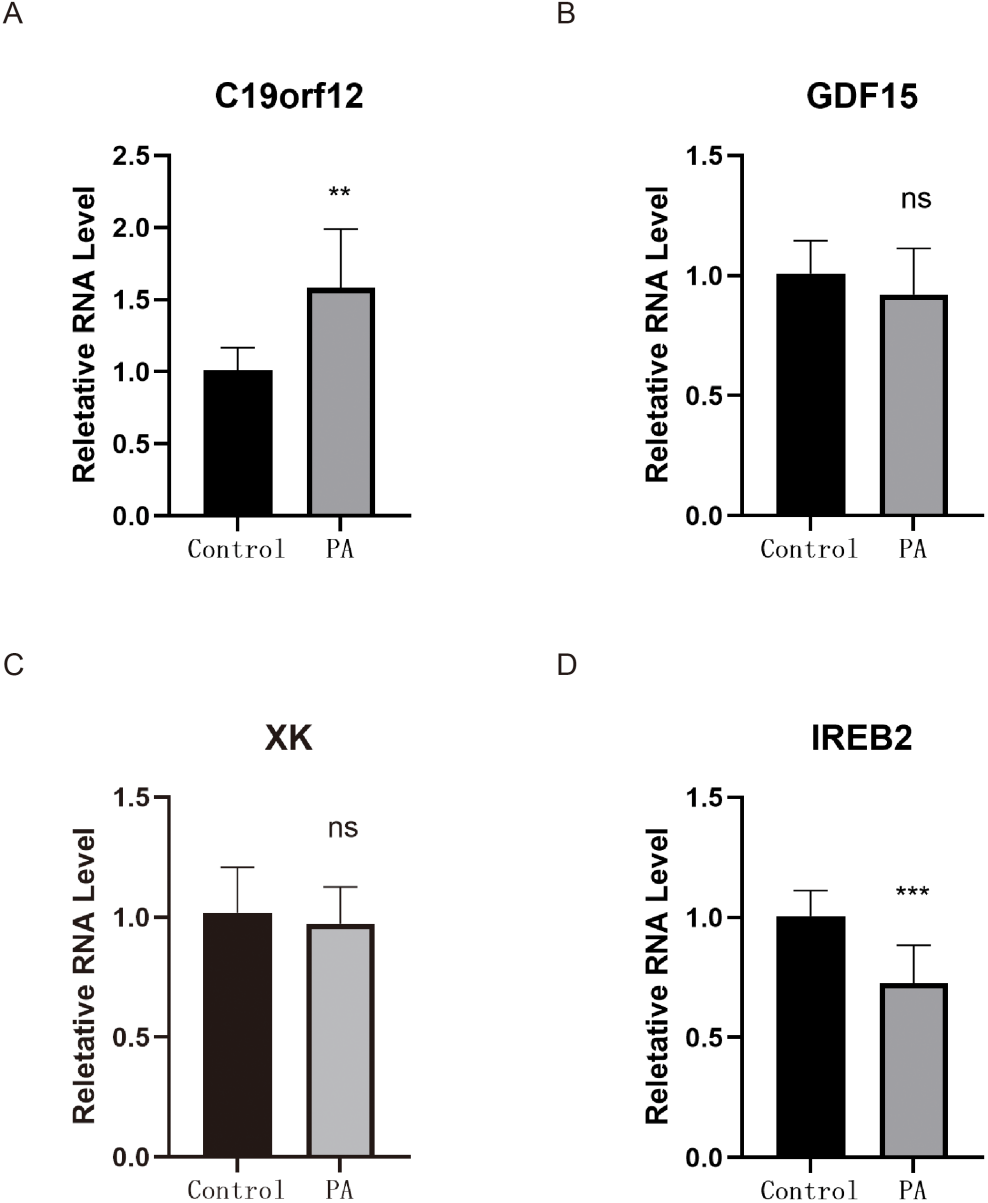
qRT-PCR validation of biomarkers in clinical samples. (A-D) Relative mRNA expression levels of *C19orf12*, *GDF15*, *XK* and *IREB2* were measured in peripheral blood samples obtained from patients with PA (n = 12) and Controls (n = 8). Data are presented as mean ± standard error of mean of relative quantification values calculated using the 2^ − ΔΔCt method with β-actin as the internal reference. Statistical significance was assessed by unpaired Student’s t-test. *p < 0.05, **p < 0.01, ***p < 0.001.

To experimentally validate the bioinformatically identified model genes, we quantified the expression levels of *C19orf12*, *GDF15*, *XK* and *IREB2* in peripheral blood samples obtained from patients with PA and healthy controls using SYBR Green-based qRT-PCR (Figs 13A–D). The results demonstrated a consistent upregulation of *C19orf12* in the PA group (mean relative quantification [RQ] range: ≈ 1.2–2.5) compared to that in the control group (mean RQ range: ≈ 0.8–1.3). In contrast, *IREB2* expression was significantly downregulated in asthma samples (mean RQ range: ≈ 0.5–0.8) compared to that in controls (mean RQ range: ≈ 0.8–1.1). The expression levels of *GDF15* and *XK* exhibited individual variability (e.g., the RQ of *GDF15* was low as 0.58 in some patients with PA); however, no consistent or statistically significant differences were observed for these genes between the two groups. These findings align partially with transcriptomic predictions and provide preliminary experimental support for the roles of *C19orf12* and *IREB2* in the pathogenesis of PA (Fig 13).

## Discussion

PA is a highly heterogeneous chronic inflammatory airway disease. Its pathogenesis and progression are influenced by multiple factors, including genetic predisposition, dysregulated immune responses, and environmental exposures [36,37]. Although research on Th2-type inflammation, IgE-mediated responses, and airway remodeling has advanced considerably [38], studies focusing on the metabolic regulation of PA remain limited. Iron metabolism, as a critical component of energy metabolism, redox balance, and immune regulation, plays a key role in various inflammatory and immune-related diseases [39]. However, the potential involvement of iron metabolism-related molecular networks in PA has not been systematically elucidated, necessitating an integrated transcriptomic analysis to explore its possible biological significance.

Based on this research background, in this study, we integrated multiple publicly available transcriptomic datasets of peripheral blood obtained from patients with PA. We combined bioinformatics analysis with machine learning methods to systematically screen DEGs related to iron metabolism and to construct an exploratory discriminant model that distinguishes patients with asthma from healthy controls. Through this analytical framework, we not only identified a set of IMRGs that may undergo dysregulation in PA but also delineated their potential functional characteristics, regulatory relationships, and immune-related patterns at a holistic level. These findings provide new molecular insights into the metabolic-immunoregulatory mechanisms underlying PA.

Differential expression analysis identified 15 IMRDGEs. Among these, *C19orf12* and *IREB2* consistently showed stable and prominent discriminatory contributions across multiple machine learning models, including logistic regression, SVM, and LASSO. This suggests their potentially central role in iron metabolism dysregulation associated with PA. *C19orf12* is closely involved in mitochondrial function maintenance and regulation of oxidative stress [40,41]. Its dysregulation can disrupt mitochondrial homeostasis and elevate ROS levels, and oxidative stress is a critical driver of amplified airway inflammation and hyperresponsiveness [42–44]. *IREB2* is an important post-transcriptional regulatory factor in iron homeostasis, which participates in the intracellular iron storage and utilization process by regulating the expression stability of IMRGs [45,46], and is abnormally expressed in various inflammatory and immune-related diseases [47,48]. These previous findings provide biologically consistent support for the recurrent identification of *C19orf12* and *IREB2* in our study and further imply their potential involvement in PA-associated inflammatory processes—possibly by mediating iron homeostasis, mitochondrial function, and oxidative stress levels.

At the pathway level, functional enrichment and GSEA revealed that the iron metabolism-related DEGs were primarily clustered in biological processes and pathways related to intracellular iron homeostasis, mitochondrial energy metabolism, redox processes, and immune-associated signaling. The pathways linked to iron homeostasis and mitochondrial function showed high consistency with the known biological roles of *C19orf12* and *IREB2*, further supporting their potential involvement in PA via a metabolism-oxidative stress axis.

Immune cells undergo significant metabolic reprogramming under inflammatory conditions [49,50], and mitochondrial dysfunction and redox imbalance can directly influence the activation status and inflammatory cytokine secretion of T cells, macrophages, and mast cells [51–56]. Integrating our findings, the aberrant enrichment of iron metabolism-related pathways suggests that metabolic regulation and immune-inflammatory processes may form an interdependent regulatory network, rather than acting through isolated pathways. Enrichment analysis indicates statistical associations between a gene set and biological processes. The results primarily serve to highlight potentially involved pathways and cannot directly infer specific molecular mechanisms or causal relationships.

To validate the reliability of bioinformatics screening results, we further performed preliminary detection of core candidate genes in peripheral blood samples using SYBR Green-based qPCR. The results demonstrated that *C19orf12* exhibited an overall upregulation trend in the asthma group, whereas *IREB2* showed downregulation, aligning with the expression patterns identified in transcriptomic analyses. These findings provide experimental support for the potential role of its dysregulation in PA. In contrast, *GDF15* and *XK* did not display stable differential expression between groups, suggesting possible variations in expression characteristics and detection sensitivity among different IMRGs in the peripheral blood. Due to limitations in sample size and source, the PCR results primarily served to confirm the expression trend consistency rather than establish definitive conclusions regarding their biological functions or clinical significance.

Immune infiltration analysis revealed differences in the relative proportions of various immune cell types between the PA and control groups, with resting memory CD4^+^ T cells and mast cells exhibiting notable alterations. A positive correlation was observed between *IREB2* expression and resting memory CD4^+^ T cell levels. These findings suggest that alterations in IMRG expression may be linked to the regulation of immune cell states. Iron metabolism and cellular metabolic status can influence T cell differentiation and functional maintenance [57–59], while mast cell activation in asthmatic inflammatory responses is also modulated by oxidative stress and the metabolic microenvironment [60–62]. It is important to emphasize that the CIBERSORT algorithm deconvolutes immune cell composition based on bulk transcriptomic data, reflecting relative trends rather than absolute cell counts. Particularly, its applicability and accuracy remain limited in peripheral blood samples. Therefore, these analytical results should be considered as supportive evidence for hypothesis generation rather than as direct proof of immune mechanisms.

Furthermore, regulatory network analysis showed that several core IMRGs may be co-regulated by multiple transcription factors and miRNAs, reflecting a certain degree of complexity at the transcriptional regulatory level. This finding provides supplementary information for understanding the multi-layered regulation of IMRGs in PA. However, as the analysis is primarily based on database predictions, further experimental studies are required for validation.

Despite employing a multi-dataset integration and multi-algorithm analysis strategy to enhance the robustness of our findings, this study has several limitations. First, all transcriptomic data were derived from publicly available GEO databases, where inherent heterogeneity exists across datasets regarding sample sources, population backgrounds, detection platforms, and clinical characteristics. Although batch effect correction methods were applied, residual heterogeneity may persist. Second, the absence of comprehensive clinical information in some datasets restricted further adjustment and stratified analyses for potential confounders such as age, sex, and disease severity. Additionally, the current research primarily relied on computational analyses and limited experimental validation, which are insufficient to draw definitive conclusions about specific molecular mechanisms or clinical applicability.

In summary, in this study, we systematically explored the potential molecular characteristics of IMRGs in PA by integrating transcriptomic data, machine learning analyses, and preliminary experimental validation. The findings suggest that an iron metabolism–immune regulatory axis may be involved in the pathogenesis of PA. These results provide a clear direction for subsequent mechanistic investigations and validation in larger cohorts. Future research should incorporate airway tissue samples, single-cell sequencing, and functional experiments to further elucidate the specific roles of the implicated genes and pathways in PA, thereby advancing a deeper understanding of the metabolic-immune regulatory mechanisms underlying this disease.

## Conclusions

This study integrates multiple transcriptome datasets and experimental data to identify key genes as candidate diagnostic markers and to support future studies on early detection and risk stratification. These findings provide new insights into the role of iron metabolism in PA pathogenesis and offer candidate biomarkers for further mechanistic research and clinical validation.

## Supporting information

S1 File S1 Table. List of IMRGs. **S2 Table.** Results of GO and KEGG Enrichment. **S3 Table.** Results of GSEA for Combined Datasets. **S4 Table.** Results of GSEA for Risk Group. **S5 Table.** mRNA-TF notes.csv. **S6 Table.** mRNA-miRNA notes. **S1 Text.** Primer Sequences.
(RAR)
